# Internet-delivered cognitive control training as a preventive intervention for remitted depressed patients: Protocol for a randomized controlled trial

**DOI:** 10.1186/s12888-015-0511-0

**Published:** 2015-06-09

**Authors:** Kristof Hoorelbeke, Lien Faelens, Jeffrey Behiels, Ernst H. W. Koster

**Affiliations:** Department of Experimental Clinical and Health Psychology, Ghent University, Henri Dunantlaan 2, B-9000 Ghent, Belgium

**Keywords:** Cognitive control, Training, Rumination, Depression, Remitted depressed, Relapse prevention, Randomized controlled trial

## Abstract

**Background:**

Preventing recurrence of depression forms an important challenge for current treatments. Cognitive control impairments often remain present during remission of depression, putting remitted depressed patients at heightened risk for new depressive episodes by disrupting emotion regulation processes. Importantly, research indicates that cognitive control training targeting working memory functioning shows potential in reducing maladaptive emotion regulation and depressive symptomatology in clinically depressed patients and at-risk student samples. The current study aims to test the effectiveness of cognitive control training as a preventive intervention in a remitted depressed sample, exploring effects of cognitive control training on rumination and depressive symptomatology, along with indicators of adaptive emotion regulation and functioning.

**Methods/design:**

We present a double blind randomized controlled design. Remitted depressed adults will complete 10 online sessions of a cognitive control training targeting working memory functioning or a low cognitive load training (active control condition) over a period of 14 days. Effects of training on primary outcome measures of rumination and depressive symptomatology will be assessed pre-post training and at three months follow-up, along with secondary outcome measure adaptive emotion regulation. Long-term effects of cognitive control training on broader indicators of functioning will be assessed at three months follow-up (secondary outcome measures).

**Discussion:**

This study will provide information about the effectiveness of cognitive control training for remitted depressed adults in reducing vulnerability for depression. Furthermore, this study will address key questions concerning the mechanisms underlying the effects of cognitive control training, will take into account the subjective experience of the patients (including a self-report measure for cognitive functioning), and explore whether these effects extend to broad measures of functioning such as Quality of Life and disability.

**Trial registration:**

This study is registered with ClinicalTrials.Gov, number NCT02407652.

## Background

Improving the effectiveness of psychotherapeutic interventions for depression forms an important challenge for depression research. That is, patients who initially respond successful to therapy, often show residual symptoms which increases the chance of recurrence of depressive episodes. Moreover, existing treatments are less effective for chronic depression [1] and not all depressive symptoms show an equal response to treatment [2]. For instance, cognitive symptoms such as impaired executive- and working memory functioning and their biological substrates often remain present although the patient is considered to be in remission (e.g., [3, 4]). Importantly, it has been suggested that reduced cognitive functioning — i.e., impaired regulation of working memory, or ‘cognitive control’ — is not merely a byproduct of depression, but places remitted depressed (RMD) patients in a distinct vulnerable position for recurrence of depression [5, 6].

Indeed, the number of previous depressive episodes shows a negative correlation with behavioral indices of cognitive control [7]. Furthermore, prospective studies suggest that self-reported cognitive control impairments [8] and their behavioral indices [9] predict the development of future depressive symptomatology. Interestingly, impaired cognitive control has typically been linked to maladaptive emotion regulation strategies such as rumination [9–11], an important cognitive vulnerability factor for depression [12]. Especially brooding — a subtype of rumination that is characterized by a passive style of moody pondering — has shown to predict the occurrence of future depressive symptomatology [13]. Importantly, prospective studies indicate that the use of maladaptive emotion regulation strategies link impaired cognitive control to the development of future depressive symptomatology in RMD [14]. Thus, via maladaptive emotion regulation cognitive control impairments convey an important risk for recurrent depression (but see [15]). Moreover, this mechanism is believed to sustain and increase biological and cognitive vulnerability for recurrent depression (for a review, see [6]).

In accordance with studies indicating plasticity of executive and working memory functioning [16], these findings have led researchers to try to remediate cognitive control impairments in depression using cognitive training tasks. In a pilot study, Siegle et al. [17] demonstrated that combining treatment as usual (TAU) with a cognitive control training (CCT) shows potential in reducing rumination as well as depressive symptomatology in a limited MDD sample. The CCT existed of the adaptive Paced Auditory Serial Addition Task (PASAT; [18]) and Well’s Attention training [19]. Furthermore, Siegle et al. [20] have extended these findings, showing long term beneficial effects of CCT by demonstrating a reduced need for outpatient services at one year follow-up. Interestingly, whereas previous studies have demonstrated the potential of a combined CCT approach [17, 20–22], other authors have shown that the training component targeting working memory functioning (the adaptive PASAT) might suffice to reduce brooding [23] and depressive symptomatology [24] in MDD patients.

These first experimental findings are in line with existing conceptual frameworks concerning the role of cognitive control and rumination in recurrent depression [6, 25], suggesting that by remediating cognitive control impairments, one might decrease cognitive vulnerability for future depression. Accordingly, Siegle et al. [20] have suggested that effects of CCT on depressive symptomatology are preceded by changes in rumination. However, to date no experimental study has directly tested this mediation effect. Furthermore, previous studies have typically explored curative effects of CCT in MDD patients whereas only more recently the preventive potential of CCT has been explored in student populations. For instance, in a single session cognitive control manipulation, Cohen et al. [26] have demonstrated that inducing cognitive control while processing negative information buffers against negative effects of a subsequent rumination induction procedure (i.e., state rumination, rumination-related sad mood). Moreover, training inhibition of emotional information has shown to reduce rumination in at-risk students [27]. Interestingly, researchers have found that the adaptive PASAT shows promise in reducing stress reactivity and rumination in response to a lab stressor directly following training and a naturalistic stressor at one month follow-up in an at-risk student sample [28]. Furthermore, decreased stress reactivity in confrontation with a lab stressor predicted lower brooding levels following confrontation with naturalistic stress (i.e., examination period). These findings suggest that CCT targeting working memory functioning shows potential as a preventive intervention for depression.

### Rational for the proposed study

Previous studies indicate that the effects of CCT are not limited to the mere reduction of current depressive symptomatology in MDD patients, but might also extend to increasing resilience in at-risk populations. However, several theoretical gaps remain to be addressed.

First, in order to fully explore the potential of CCT targeting working memory functioning in reducing (cognitive) vulnerability for depression, a test of training effects in a RMD sample would be desirable. That is, RMD patients form a high-risk group for developing future depressive episodes [29] and prospective studies indicate that impaired cognitive control forms an important vulnerability factor in RMD [14]. Second, from a theoretical stance it would be interesting to explore the proposed mediational pathway from effects of CCT on rumination to reduced future depressive symptomatology. Third, with the exception of Siegle et al. [20] who explored effects of CCT on outpatient service use, previous studies have limited their scope to exploring effects of CCT on rumination and depressive symptomatology. We aim at extending previous findings by also exploring effects of CCT on adaptive emotion regulation as well as broader indicators of (dis-)functioning such as experienced disability, experienced remission from depression, and Quality of Life. Furthermore, we are not only interested in change in behavior indices of cognitive control, but also in the clinical experience of RMD patients concerning these cognitive factors (e.g., self-report measures of executive- and working memory functioning). Finally, in order to reduce sources of bias in exploring the potential of CCT as a preventive intervention, a rigid methodological approach — i.e., a double-blind randomized controlled trial (RCT) — is required.

## Method

### Design

**Fig. 1:**
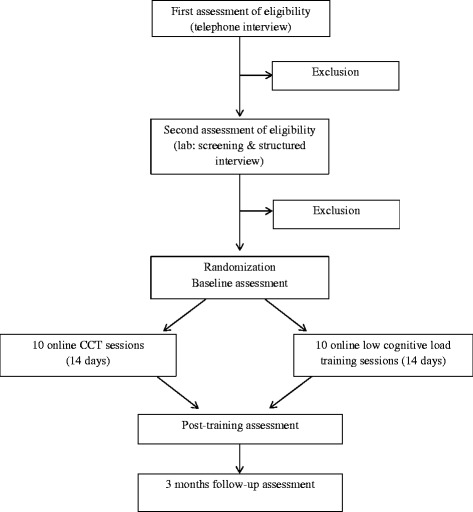
Study flowchart

We present a 2 (Condition) x 3 (Time) double blind, randomized controlled design. Adult RMD participants will be randomly allocated to either an online CCT intervention targeting working memory functioning or a low cognitive load training (active control condition). Both groups will perform 10 online training sessions over a period of 14 days, flanked by pre- and post-training lab assessments. Participants will return to the lab for a final assessment at three months follow-up (see Fig. 1 for an overview of the design). This study has been approved by the local ethical committee of the Faculty of Psychology and Educational Sciences of Ghent University and was registered with ClinicalTrials.gov, number NCT02407652.

### Participants

#### Inclusion and exclusion criteria

To be eligible for participation to this study, participants aged between 23 and 65 should report a history of depression (at least one prior depressive episode) and report stable (partial) remission (≥6 months). Consequently, participants should not meet criteria for a current depressive episode before starting training as assessed by the Mini-International Neuropsychiatric Interview (MINI; [30]). However, they should meet the criteria for a previous episode. The depressive episode should not have occurred in the context of a bipolar disorder. Neither should the participant report a history of psychosis, excessive substance abuse, or report experiencing cognitive impairments due to brain injury. A history of other comorbid disorders is allowed — yet these should not lead to current impairments — in order to increase the clinical relevance and validity of our study. Therapeutic maintenance contact (with a frequency less than once per three weeks) and use of antidepressant medication is allowed and will be registered. Importantly, antidepressant medication should be kept at a constant level throughout the course of the study.

#### Recruitment

RMD participants will be recruited using advertisements in popular (online) magazines and newspapers as well as flyers that were placed in 106 local drugstores (Ghent area). Furthermore, drawing on an existing database, 23 potentially interested participants will be contacted based on their interest in a prior prospective study of our lab (at that point, all of these participants had given their permission to be re-contacted in case a related study was planned). After having expressed interest in this study (i.e., by phone or e-mail), participants will be contacted by phone to provide further information and to screen eligibility based on a selection of relevant questions of the MINI screening version. To screen whether participants show a history of depression, we will ask questions concerning current and previous depressive symptoms, and collect information concerning the amount of episodes and past as well as current treatment. Furthermore, we will check whether (professional or similar) activities were resumed following the last depressive episode. If the participant seems eligible and is interested in participating in the study, he or she will be invited to the lab for a structured clinical interview (MINI). In the lab, the MINI screening version will be used to check for indicators of currently present comorbid disorders and — if this proves to be necessary — will be followed by the corresponding parts of the MINI interview to allow to control for presence of comorbid disorders. The parts related to MDD will be fully assessed to assure that participants do not meet the criteria for MDD before entering the study. Meeting eligibility criteria will allow the participant to enroll in the study, starting immediately with randomization over one of both conditions and the baseline assessment.

### Measures

#### Primary outcome measures

Rumination and depressive symptomatology form our primary outcome measures. *Rumination* will be assessed using the Ruminative Response Scale (RRS; [13, 31]). This 22-item questionnaire provides a total rumination score (range: 22 – 88), as well as Brooding and Reflection subscale scores (range: 5 – 20). Brooding is characterized by a passive style of moody pondering and is the most maladaptive form of depressive rumination [13, 32]. *Depressive symptomatology* will be assessed using the 21-item (range: 0 – 63) Beck Depression Inventory (BDI-II; [33, 34]). Both primary outcome measures exhibit adequate psychometric properties [13, 33, 34].

#### Secondary outcome measures

Adaptive emotion regulation and indicators of RMD functioning will be our secondary outcome measures. *Adaptive emotion regulation* will be assessed using the five adaptive subscales of the Cognitive Emotion Regulation Questionnaire (CERQ; [35]). The four less adaptive strategies (self-blame, rumination, catastrophizing, and blaming others) can be used as a second, alternative measure for maladaptive emotion regulation (range: 4 – 20).

*Functioning* will be operationalized by indices of disability, quality of life, resilience, and remission from depression. *Disability* will be assessed using the self-report version of the World Health Organization Disability Assessment Schedule 2.0 (WHODAS 2.0; [36]), consisting of 36 items. This measure is based on the conceptual framework of the International Classification of Functioning (ICF) and provides indicators of overall functioning and six specific domains of functioning (Cognition, Mobility, Self-care, Getting along, Life activities, and Participation). Furthermore, the questionnaire provides an estimate of the amount of days in the past month during which the difficulties (a) were present, (b) prevented the participant from performing his/her daily activities or work, or (c) formed a source of reduced functioning. *Quality of life* will be assessed using the depression-specific 34-item (range: 0 – 34) Quality of Life in Depression Scale (QLDS; [37, 38]. *Resilience* will be assessed using the Resilience Scale (RS; [39, 40]). We will use the 25-item version of the RS using four point Likert-scales (range: 25 – 100). Finally, self-reported *remission from depression* will be assessed using the 41-item Remission of Depression Questionnaire (RDQ; [41]; Dutch translation: Peeters et al.: RDQ-NL, unpublished document) for which a high score is indicative for more psychopathology (range: 0 – 82).

#### Manipulation check, training process and cognitive transfer measures

As a manipulation check and process measure, *training task performance* will be assessed in both conditions using median inter stimulus interval (ISI) levels per training session. Furthermore, as a process measure of effects of completing an online training session, mood (‘energetic’, ‘tense’, ‘frustrated’, ‘sad’, ‘happy’) will be assessed using visual analogue scales (VAS; 1 – 100). The extent to which participants have experienced negative thoughts and stress throughout the training session will also be assessed using VAS, along with experienced task competence (‘During the task I felt as if I was doing great’). It has been suggested that training cognitive control in a frustrating task-context — and thus, eliciting low levels of negative affect while training — contributes to the beneficial effects of CCT [28]. These process measures allow to explore the mechanism underlying CCT. Furthermore, we will use the Credibility/Expectancy Questionnaire (CEQ; [42]; Dutch translation: Godfrin et al.: CEQ-NL, unpublished document) to check for baseline group differences in treatment credibility/expectancy and to check for successful blinding of participants (post-training). Moreover, we will monitor intake of antidepressants and other forms of therapy as well as stressful life events that might influence our findings. For the latter, we will use the List of Threatening Experiences (LTE; [43, 44]).

Close transfer to *cognitive control* will be assessed using the non-adaptive PASAT [18]. During this task, participants are presented with a practice phase consisting of 10 trials, followed by a test phase, consisting of three blocks with increasing difficulty (ISI block 1 = 3000 ms; ISI block 2 = 2000 ms; ISI block 3 = 1500 ms), each containing 60 trials. Furthermore, we will include the Behavior Rating Inventory of Executive Function Adult version (BRIEF-A; [45]) as a self-report measure to assess experienced cognitive control. This 75-item self-report questionnaire provides several estimates of executive and working memory functioning (e.g., inhibition, shifting, emotional control, working memory).

### Interventions

Participants will either be subjected to an online CCT targeting working memory functioning (the adaptive PASAT) or a closely matched low cognitive load training. This allows to rule out motivational aspects of performing an adaptive computer task online. The tasks will be presented in-browser, using a Millisecond software Web license. Both training groups will be asked to perform 10 sessions of 400 trials (which takes 20 min per session at an average inter stimulus interval (ISI) of 3000 ms), providing similar learning experiences in both conditions. Prior to training, both groups will receive oral and written psycho-education concerning cognitive control training (based on the protocol of [17]) in order to enhance task engagement. This is important as previous work indicates that task engagement forms an important predictor of response to CCT [20]. Importantly, no explicit information will be given about the to be expected results. Furthermore, participants will receive an automated text message on a daily basis to prevent attrition during the training period (using SurveySignal software; [46]).

#### Cognitive control training condition

We will use an adaptive version of the PASAT [17, 18] to train participants’ cognitive control in the CCT condition. Participants will be presented with a continuous stream of auditory digits (1–9) and are instructed to immediately respond to the sum of the last two heard digits by clicking the corresponding response buttons (1–18). The speed of number presentation is adapted based on participants’ performance in order to train cognitive control in a frustrating task context. Participants begin each session with a 3000 ms ISI, which is reduced by 100 ms following every four consecutive correct responses, increasing task difficulty. Following every four incorrect responses the ISI increases with 100 ms, reducing task difficulty. Throughout each session participants are presented with their current ISI and amount of consecutive correct and incorrect responses. Participants’ responses and response times are being measured. In line with previous training studies the median ISI per session will be used as an indicator of ones performance during the training sessions.

#### Active control condition

In the active control condition, participants will be presented with a low cognitive load version of the adaptive PASAT. This training task shows high resemblance to the adaptive PASAT concerning stimuli, responses, modification of task difficulty, and evaluation of session performance. However, in this low cognitive load version of the adaptive PASAT participants are instructed to immediately respond to the last heard digit instead of mentally manipulating the content in working memory (i.e., instead of responding to the sum of the last two heard digits as in the CCT condition). To better resemble the response options of the adaptive PASAT, participants in the active control condition are presented auditory stimuli ranging from 1–18.

### Sample size

We are the first to explore effects of CCT targeting working memory functioning in a RMD sample which makes it impossible to provide an exact estimate of effect size for the main outcome measure in this sample. However, previous work on MDD patients has yielded an effect size of *ɳ*^*2*^ = .19 for brooding [20], whereas work with at-risk undergraduate students revealed an effect size of *ɳ*^*2*^ = .11 in confrontation with naturalistic stress [28]. Given that this study will use an at-risk sample (RMD), we will base estimations of sample size on the latter effect size. In order to be able to detect a similar effect over two time points with *α* = .05 and 1-β = .80, the total sample size should at least be 68 (*n* CCT = 34, *n* active control = 34). We will stop recruiting once 68 participants have entered the training phase.

### Randomization

Upon entering the study, participants will receive a sealed envelope containing an exterior subject number that will be used for registration purposes during the assessment sessions in the lab (baseline, post-training, and follow-up). The envelope will contain a training manual, an URL that directs participants to the online training task, and a personal training task identification code that should be used while performing the ten online training sessions at home. Prior to the study, an independent researcher will randomly link the training task identification codes to the subject numbers that will be used in the lab using an automated randomization program (RandList; randomisation.eu). This researcher will prepare the envelopes and keep a list of the linked subject numbers and training session identification codes in a locked closet at the office and a copy at home for safe keeping. Based on the training task identification codes, participants will either perform the CCT or low cognitive load training.

### Blinding

We present a double blind RCT design. Prior to the randomization procedure, the independent researcher will reset the online training task so that even-numbered training task identification codes will redirect the participants to one condition (CCT or active control), whereas odd-numbered training task identification codes will redirect the participants to the other condition. The researchers of this study will not be aware of the training task identification codes (these are randomly generated and presented in a sealed envelope) or the link between even- or odd-numbered identification codes and training condition. Furthermore, participants will be instructed not to share details concerning the content of the training task or the personal training task identification code with the researchers.

During data-analysis, the researchers will remain blind of training task condition by separating (a) analysis of training task performance and process measures (based on even- or odd-numbered training task identification codes) from (b) analysis of training effects on the outcome measures. Concerning the latter, the independent researcher will provide the researchers with a list grouping the subject numbers — used during the lab sessions — in two non-informative conditions following completion of data-collection. Importantly, at this point (lab) subject numbers will not be linked to the personal training task identification codes. This allows blind evaluation of training effects. The blinding will only be broken for the more explorative analyses linking training task process measures with the outcome measures. Furthermore, we will use the CEQ-data to check for successful blinding of participants.

### Analysis

In line with Consolidated Standards of Reporting Trials (CONSORT; [47]), we will use intention-to-treat (ITT) analysis to test effects of CCT on primary and secondary measures post-training and at follow-up. Missing data will be handled using the Last-Observation-Carried-Forward (LOCF) method. Effects of CCT will be tested using Repeated Measures, analysis of variance (ANOVA), or covariance (ANCOVA) with follow-up *t*-tests. Exploratory analysis will take into account potential moderators of training effects such as variability in baseline depressive symptomatology and cognitive control. ITT might not necessarily apply to the exploratory analyses such as analysis of process measures of training. As secondary analysis, we will also perform completers-only analyses. Explorative within-group mediation analysis will be performed using a stepwise regression approach [48] and the Preacher and Hayes [49] bootstrapping method.

### Procedure

Eligibility will be assessed by a clinical psychologist. Participants will first undergo a telephone screening to assess eligibility (see Fig. 1). Second, potential participants will be invited to the lab where eligibility will be further assessed using the MINI. After giving informed consent, eligible participants will be randomized and the baseline assessment will take place (see Table 1). At baseline (Time 1), the behavior measure for cognitive control will be completed followed by the self-report measure for cognitive control (BRIEF-A). Next, participants will complete the other self-report questionnaires (primary outcome measures: BDI-II, RRS; secondary outcome measures: CERQ, QLDS, WHODAS 2.0, RS, RDQ) and will receive psycho-education concerning cognitive training for depression and practical information about the intervention. Participants will be instructed to complete 10 training sessions during a period of 14 days following the baseline assessment and will be asked to perform only one session a day. At the end of the baseline assessment session, the CEQ will be administered and participants’ telephone number will be registered using SurveySignal software. During the 14-days period of online training, participants will receive a daily automated text message reminding them to complete the training. Each training session will consist of 400 trials of the adaptive PASAT or a low cognitive load training and will include assessments of affect and worrying throughout and following training. Upon completing training, participants will return to the lab for the post-training assessment (Time 2) during which direct effects of CCT on cognitive control and the primary outcome measures and adaptive emotion regulation will be assessed. At the end of the post-training session the CEQ will be administered to rule out group differences in expectancy and credibility of the intervention. Finally, participants will return to the lab at three months follow-up (Time 3) during which long-term effects on cognitive control and the primary and secondary outcome measures (including indicators of functioning) will be assessed. At each time point we will assess stressful life events (LTE), intake of antidepressants and other forms of therapy. Upon completion of the follow-up assessment session, participants will receive reimbursement (€75) followed by a partial written and oral debriefing. Importantly, participants will only receive feedback concerning their condition following processing of the data of the total sample. If CCT shows to have beneficial effects in RMD, participants from the active control condition will be offered the chance to perform the CCT online.

**Table 1:** Schedule of measures

Instrument	Telephone screening	Baseline	Online training	Post-training (2 weeks)	Follow-up (3 months)
Inclusion criteria interview	X				
MINI Screen and structured interview		X			
Process measures of training task experience			X		
Process measures of training task performance (ISI)			X		
Credibility and expectancy of treatment (CEQ)		X		X	
Stressful life events (LTE)		X		X	X
Self-reported use of antidepressants and other forms of therapy		X		X	X
Cognitive control (non-adaptive PASAT / transfer task)		X		X	X
Self-reported cognitive control (BRIEF-A)		X		X	X
Depressive symptomatology (BDI-II)		X		X	X
Depressive rumination (RRS)		X		X	X
Cognitive emotion regulation (CERQ)		X		X	X
Quality of Life (QLDS)		X			X
Disability (WHODAS 2.0)		X			X
Resilience (RS)		X			X
Remission from depression (RDQ)		X			X

## Discussion

Prevention of recurrent depression is an important target for interventions. Previous findings indicate that CCT shows potential in reducing depressive symptomatology and rumination in MDD as well as cognitive vulnerability in at-risk undergraduate students. To test the potential of CCT as a preventive intervention for depression, the present study aims to test the effectiveness of CCT in a RMD sample. We will test whether CCT targeting working memory functioning — as compared to a low cognitive load training — can be used to reduce vulnerability for depression over a 3.5 months period. We hypothesize that CCT will have beneficial effects on primary outcome measures depressive rumination (i.e., brooding) and depressive symptomatology and hope to see these findings extend to adaptive emotion regulation and long-term functioning (secondary outcome measures).

This double blind RCT study forms a first test of the potential of CCT as a preventive intervention for depression in RMD. Furthermore, these findings will be informative to the literature as several exploratory questions will be addressed in order to further elucidate the role of cognitive control in vulnerability for depression. First, we will explore whether effects of CCT on depressive symptomatology are mediated by rumination. Second, we will explore whether effects of CCT extend to measures of adaptive emotion regulation and indices of functioning such as quality of life and disability. Third, in order to further elucidate the mechanisms involved during CCT, we could explore how process measures of CCT relate to effects of training.

Overall, this study will further enhance the knowledge on the role of cognitive control in emotion regulation and vulnerability for depression. This study forms a first step in testing the effectiveness of CCT targeting working memory functioning as a preventive intervention for (recurrent) depression. If these first results show to be promising, future work should focus on replicating the effects of CCT and exploring how this preventive intervention could best be implemented.

### Trial status

We are currently recruiting RMD patients for this study. The study entered the data collection phase in April 2015.

## Abbreviations

ANCOVA: Analysis of covariance
ANOVA: Analysis of variance
BDI-II: Beck Depression Inventory 2^nd^ edition
BRIEF-A: Behavior Rating Inventory of Executive Function Adult version
CCT: Cognitive control training
CEQ: Credibility/Expectancy Questionnaire
CERQ: Cognitive Emotion Regulation Questionnaire
CONSORT: Consolidated Standards of Reporting Trials
ICF: International Classification of Functioning
ISI: Inter stimulus interval
ITT: Intention-to-treat
LOCF: Last-Observation-Carried-Forward
LTE: List of Threatening Experiences
MDD: Major depressive disorder
PASAT: Paced Auditory Serial Addition Task
QLDS: Quality of Life in Depression Scale
RCT: Randomized controlled trial
RDQ: Remission of Depression Questionnaire
RMD: Remitted depressed
RRS: Ruminative Response Scale
RS: Resilience Scale
WHODAS: World Health Organization Disability Assessment Schedule 2.0

## References

[1] Cuijpers P, Andersson G, Donker T, van Straten A (2011). Psychological treatment of depression: results of a series of meta-analyses. Nord J Psychiatr.

[2] Millan MJ, Agid Y, Brüne M, Bullmore ET, Carter CS, Clayton NS, Connor R, Davis S, Deakin B, DeRubeis RJ (2012). Cognitive dysfunction in psychiatric disorders: characteristics, causes and the quest for improved therapy. Nat Rev Drug Discov.

[3] Vanderhasselt M-A, De Raedt R (2009). Impairments in cognitive control persist during remission from depression and are related to the number of past episodes: An event related potentials study. Biol Psychol.

[4] Xu GY, Lin KG, Rao DP, Dang YM, Ouyang HY, Guo YB, Ma JX, Chen JC (2012). Neuropsychological performance in bipolar I, bipolar II and unipolar depression patients: a longitudinal, naturalistic study. J Affect Disord.

[5] Gotlib IH, Joormann J, Nolen-Hoeksema S, Cannon TD, Widiger T (2010). Cognition and Depression: Current Status and Future Directions. Annu Rev Clin Psychol.

[6] De Raedt R, Koster EHW (2010). Understanding vulnerability for depression from a cognitive neuroscience perspective: A reappraisal of attentional factors and a new conceptual framework. Cogn Affect Behav Neurosci.

[7] Harvey PO, Le Bastard G, Pochon JB, Levy R, Allilaire JF, Dubois B, Fossti P (2004). Executive functions and updating of the contents of working memory in unipolar depression. J Psychiatr Res.

[8] Letkiewicz AM, Miller GA, Crocker LD, Warren SL, Infantolino ZP, Mimnaugh KJ, Heller W (2014). Executive function deficits in daily life prospectively predict increases in depressive symptoms. Cogn Ther Res.

[9] Zetsche U, Joormann J (2011). Components of interference control predict depressive symptoms and rumination cross-sectionally and at six months follow-up. J Behav Ther Exp Psychiatry.

[10] De Lissnyder E, Koster EHW, Goubert L, Onraedt T, Vanderhasselt MA, De Raedt R (2012). Cognitive control moderates the association between stress and rumination. J Behav Ther Exp Psychiatry.

[11] Joormann J, Gotlib IH (2010). Emotion regulation in depression: relation to cognitive inhibition. Cogn Emot.

[12] Nolen-Hoeksema S, Wisco BE, Lyubomirsky S (2008). Rethinking rumination. Perspect Psychol Sci.

[13] Treynor W, Gonzalez R, Nolen-Hoeksema S (2003). Rumination reconsidered: a psychometric analysis. Cogn Ther Res.

[14] Demeyer I, De Lissnyder E, Koster EHW, De Raedt R (2012). Rumination mediates the relationship between impaired cognitive control for emotional information and depressive symptoms: a prospective study in remitted depressed adults. Behav Res Ther.

[15] Aker M, Harmer C, Landro NI (2014). More rumination and less effective emotion regulation in previously depressed women with preserved executive functions. BMC Psychiatry.

[16] Klingberg T (2010). Training and plasticity of working memory. Trends Cogn Sci.

[17] Siegle GJ, Ghinassi F, Thase ME (2007). Neurobehavioral therapies in the 21st century: summary of an emerging field and an extended example of cognitive control training for depression. Cogn Ther Res.

[18] Gronwall D (1977). Paced auditory serial-addition task: a measure of recovery from concussion. Percept Mot Skills.

[19] Wells A (2000). Emotional disorders and metacognition innovative cognitive therapy.

[20] Siegle GJ, Price RB, Jones NP, Ghinassi F, Painter T, Thase ME (2014). You gotta work at it: pupillary indices of task focus are prognostic for response to a neurocognitive intervention for rumination in depression. Clinical Psychological Science.

[21] Calkins AW, McMorran KE, Siegle GJ, Otto MW. The effects of computerized cognitive control training on community adults with depressed mood. Behav Cognit Psychther. in press. 10.1017/S1352465814000046 24589123

[22] Segrave RA, Arnold S, Hoy K, Fitzgerald PB (2014). Concurrent cognitive control training augments the antidepressant efficacy of tDCS: a pilot study. Brain Stimul.

[23] Vanderhasselt M-A, De Raedt R, Namur V, Lotufo PA, Bensenor IM, Boggio PS, Brunoni AR. Transcranial electric stimulation and neurocognitive training in clinically depressed patients: A pilot study of the effects on rumination. Prog Neuropsychopharmacol Biol Psychiatry. in press, 57:93-99. 10.1016/j.pnpbp.2014.09.015 25455589

[24] Brunoni AR, Boggio PS, De Raedt R, Bensenor IM, Lotufo PA, Namur V, Valiengo LCL, Vanderhasselt MA (2014). Cognitive control therapy and transcranial direct current stimulation for depression: a randomized, double-blinded, controlled trial. J Affect Disord.

[25] Joormann J, D’Avanzato C (2010). Emotion regulation in depression: examining the role of cognitive processes. Cogn Emot.

[26] Cohen N, Mor N, Henik A. Linking executive control and emotional response: A training procedure to reduce rumination. Clinical Psychological Science. in press.

[27] Daches S, Mor N (2014). Training ruminators to inhibit negative information: a preliminary report. Cogn Ther Res.

[28] Hoorelbeke K, Koster EHW, Vanderhasselt MA, Callewaert S, Demeyer I. The influence of cognitive control training on stress reactivity and rumination in response to a lab stressor and naturalistic stress. Behav Res Ther. in press. 10.1016/j.brat.2015.03.010 25841177

[29] Burcusa SL, Iacono WG (2007). Risk for recurrence in depression. Clin Psychol Rev.

[30] Sheehan DV, Lecrubier Y, Sheehan KH, Amorim P, Janavs J, Weiller E, Herqueta T, Baker R, Dunbar GC (1989). The Mini-International Neuropsychiatric Interview (MINI): The development and validation of a structured diagnostic psychiatric interview for DSM-IV and ICD-10. J Clin Psychiatry.

[31] Nolen-Hoeksema S, Morrow J (1991). A prospective study of depression and posttraumatic stress symptoms after a natural disaster - The 1989 Loma-Prieta earthquake. J Pers Soc Psychol.

[32] Joormann J, Dkane M, Gotlib IH (2006). Adaptive and maladaptive components of rumination? Diagnostic specificity and relation to depressive biases. Behav Ther.

[33] Beck AT, Steer RA, Brown GK (1996). Manual for Beck Depression Inventory II.

[34] Van der Does AJW (2002). BDI-II-NL. Handleiding: De Nederlandse versie van de Beck Depression Inventory - 2nd edition.

[35] Garnefski N, Kraaij V, Spinhoven P (2001). Negative life events, cognitive emotion regulation and emotional problems. Pers Individ Differ.

[36] Ustün T, Kostanjsek N, Chatterji S, Rehm J (2010). Measuring health and disability: Manual for WHO Disability Assessment Schedule (WHODAS 2.0).

[37] Hunt SM, McKenna SP (1992). The QLDS: A scale for the measurement of quality of life in depression. Health Policy.

[38] Tuynman-Qua H, de Jonghe F, McKenna SP (1997). Quality of Life in Depression Scale (QLDS). Development, reliability, validity, responsiveness and application. Eur Psychiatry.

[39] Wagnild GM, Young HM (1993). Development and psychometric evaluation of the resilience scale. J Nurs Meas.

[40] Portzky M (2008). RS-nl: Resilience Scale - Nederlandse versie.

[41] Zimmerman M, Martinez JH, Attiullah N, Friedman M, Toba C, Boerescu DA, Ragheb M (2013). A new type of scale for determining remission from depression: the Remission from Depression Questionnaire. J Psychiatr Res.

[42] Devilly GJ, Borkovec TD (2000). Psychometric properties of the credibility/expectancy questionnaire. J Behav Ther Exp Psychiatry.

[43] Rosmalen JGM, Bos EH, de Jonge P (2012). Validation of the Long-term Difficulties Inventory (LDI) and the List of Threatening Experiences (LTE) as measures of stress in epidemiological population-based cohort studies. Psychol Med.

[44] Brugha TS, Cragg D (1990). The List of Threatening Experiences: the reliability and validity of a brief life events questionnaire. Acta Psychiatr Scand.

[45] Roth RM, Lance CE, Isquith PK, Fischer AS, Giancola PR (2013). Confirmatory factor analysis of the Behavior Rating Inventory of Executive Function-Adult version in healthy adults and application to attention-deficit/hyperactivity disorder. Arch Clin Neuropsychol.

[46] Hofmann W, Patel PV. SurveySignal: a convenient solution for experience sampling research using participants’ own smartphones. Soc Sci Comput Rev. in press.

[47] Moher D, Hopewell S, Schulz KF, Montori V, Gotzsche PC, Devereaux PJ, Elbourne D, Egger M, Altman DG (2010). CONSORT 2010 Explanation and elaboration: Updated guidelines for reporting parallel group randomised trials. Br Med J.

[48] Baron RM, Kenny DA (1986). The moderator-mediator variable distinction in social psychological research - Conceptual, strategic, and statistical considerations. J Pers Soc Psychol.

[49] Preacher KJ, Hayes AF (2004). SPSS and SAS procedures for estimating indirect effects in simple mediation models. Behav Res Methods Instrum Comput.

